# Deriving and validating a protocol to determine the need for prophylactic peritoneal dialysis in neonates after cardiopulmonary bypass surgery

**DOI:** 10.1007/s00467-024-06327-3

**Published:** 2024-03-04

**Authors:** Tennille N. Webb, Santiago Borasino, Kristal M. Hock, Inmaculada Aban, Daryl Ingram, Kara Short, Robert Dabal, David Askenazi

**Affiliations:** 1 Pediatrics/Pediatric Nephrology, University of Alabama at Birmingham/Children’s of Alabama, Birmingham, AL USA; 2 Pediatrics/Pediatric Cardiac Intensive Care Unit, University of Alabama at Birmingham/Children’s of Alabama, Birmingham, AL USA; 3 School of Public Health, University of Alabama at Birmingham, Birmingham, AL USA; 4 Pediatrics/Pediatric Nephrology, Children’s of Alabama, Birmingham, AL USA; 5 Pediatrics/Pediatric Cardiothoracic Surgery, University of Alabama at Birmingham/Children’s of Alabama, Birmingham, AL USA

**Keywords:** Peritoneal dialysis, Cardiopulmonary bypass, Neonates, Acute kidney injury

## Abstract

**Background:**

Prophylactic peritoneal dialysis (PD) in neonates undergoing cardiopulmonary bypass (CPB) is safe and improves outcomes. We sought to (1) derive the pre-operative characteristics of neonates who are most likely to benefit from PD after CPB and (2) validate a new prophylactic PD protocol based on our retrospective analysis.

**Methods:**

First, we retrospectively evaluated neonates requiring cardiac surgery with CPB from October 2012 to June 2016. We categorized neonates as those who “needed PD” and those who “did not need PD” based on prior experience with neonates requiring kidney support therapy. Pre-operative serum creatinine ≥ 0.8 mg/dL, pre-operative weight ≤ 2.5 kg, or having an open chest post-operatively were independently associated with “needed PD.” Next, beginning in March 2019, we implemented a new prophylactic PD protocol in which only those who met at least one of the three criteria derived in the retrospective analysis had a PD catheter placed in the OR.

**Results:**

In Era 2, after the implementation of a new prophylactic PD protocol, 100% of neonates in the “needed PD” group had a PD catheter placed in the OR, which was more than in the prior era (Era 1 = 86.6%) (*p* = 0.05). Only 26.1% in the “did not need PD” group had a PD catheter placed in the OR which was less than in the prior era (Era 1 = 50.6%) (*p* < 0.01).

**Conclusions:**

We successfully developed and implemented an evidence-based prophylactic PD protocol that has improved our ability to provide prophylactic PD in neonates after CPB.

**Graphical Abstract:**

**Figure:**
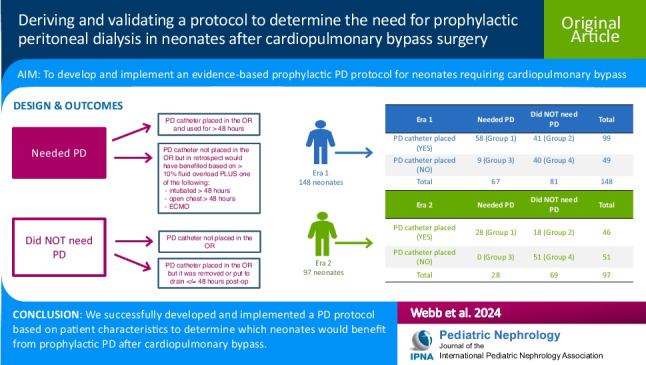
A higher resolution version of the Graphical abstract is available as Supplementary information

**Supplementary Information:**

The online version contains supplementary material available at 10.1007/s00467-024-06327-3.

## Introduction

Peritoneal dialysis (PD) is the common modality of kidney support therapy (KST) in smaller patients, especially neonates. In neonates who undergo cardiopulmonary bypass (CPB), PD has been associated with improved outcomes; however, there is no consensus on which neonates would most benefit from prophylactic PD [1–3]. Several studies have demonstrated prophylactic PD can improve outcomes, including better fluid balance, fewer electrolyte abnormalities, shorter duration of mechanical ventilation, and decreased length of ICU stay [3–5]. When placed in the operating room (OR), PD is safe with very few adverse events [3, 6, 7]. Prophylactic PD has been compared to the use of furosemide for fluid overload in infants after cardiac surgery. Those who received furosemide were more likely to have worse outcomes, including 10% fluid overload [4].

Although PD after CPB may improve clinical outcomes, the practice variation across and within centers varies. Some programs place a PD catheter in the OR for almost all neonates after CPB, while others only place them when fluid overload develops days after surgery. Evidence-based protocols for determining who will most benefit from early PD can optimize care for those who need PD, and reduce cost/nursing time and complications from those who do not need PD.

To improve the ability to provide PD only to those neonates who need it, we performed a retrospective analysis to determine the patient characteristics that delineate which neonates would most benefit from prophylactic PD after CPB. In May 2019, we implemented a protocol for PD catheter placement in the OR based on the derived characteristics from our retrospective analysis. Then, we evaluated the effectiveness of our new protocol and compared our findings to our prior protocol.

## Materials and methods

### Population and study details for Era 1

We performed a single-center retrospective analysis of neonates with congenital heart disease who required cardiac surgery with CPB from October 2012 to June 2016 at Children’s of Alabama. Inclusion criteria included all neonates less than 30 days of age who required CPB during cardiac surgery. Exclusion criteria included neonates who were started on KST before cardiac surgery.

### Defining the need for PD

We classified neonates as those who “needed PD” and those who “did not need PD” based on clinical criteria that we determined, which have previously been shown to be associated with poor clinical outcomes and clinical acumen. Table 1 outlines how neonates were classified into “needed PD” vs. “did not need PD” groups. Neonates who “needed PD” were those who either had a PD catheter placed in surgery that was used for more than 48 h or were those who did not have a PD catheter placed in surgery but in retrospect would have benefitted from PD (> 10% fluid balance positive plus one of the following: intubated for more than 48 h, had an open chest for more than 48 h, and/or required extracorporeal membrane oxygenation (ECMO)). Alternatively, neonates defined as those who “did not need PD” had a PD catheter placed during surgery but either (a) the PD catheter was removed or put to drain ≤ 48 h post-operatively or (b) did not have a PD catheter placed during surgery and did not meet any of the specified criteria for benefitting from PD based on our above criteria.

**Table 1:** Defining the need for PD

Condition 1 (Needed PD)	Condition 2 (Did not need PD)
• PD catheter placed in the OR and used for > 48 h	• PD catheter not placed in the OR
• PD catheter not placed in the OR but in retrospect would have benefited based on > 10% fluid overload PLUS one of the following:	• PD catheter placed in the OR but it was removed or put to drain ≤ 48 h post-op
○ Intubated > 48 h	
○ Open chest > 48 h	
○ ECMO	

*PD* Peritoneal dialysis, *OR* Operating room, *ECMO* Extracorporeal membrane oxygenation

### Population and methods for Era 2

In May 2019, we implemented a new protocol whereby only neonates who met one of the three criteria we found in the first era had PD catheters placed in the OR followed by prophylactic PD. We performed a prospective observational study of neonates who underwent CPB between March 2019 and March 2021 and did not require KST before CPB.

### Perioperative characteristics

Perioperative characteristics were selected based on previously reported studies and included age at the time of surgery, sex, birthweight, gestational age, the Society of Thoracic Surgeons-European Association for Cardio-Thoracic Surgery (STAT) score, CPB time, lowest pre-operative SCr, pre-operative weight, and open chest post-operatively.

### Participant allocation

Neonates were classified into four groups based on the 2 × 2 table below for the variables of whether or not a PD catheter was placed in the OR and whether they “needed PD” vs. “did not need PD.” The cohort was divided into the following 4 groups (Table 2):

- Group 1 were those who had a PD catheter placed and “needed PD.”
- Group 2 were those who had a PD catheter placed and “did not need PD.”
- Group 3 were those who did not have a PD catheter placed and “needed PD.”
- Group 4 were those who did not have a PD catheter placed and “did not need PD.”

**Table 2:** 2 × 2 table for Era 1 need for PD by PD placement

PD catheter placed	Needed PD	Did not need PD	Total
PD catheter placed (yes)	58 (group 1)	41 (group 2)	99
PD catheter placed (no)	9 (group 3)	40 (group 4)	49
Total	67	81	148

*PD* Peritoneal dialysis

These four groups were finally categorized into those who “needed PD” (groups 1 and 3 combined) and those who “did not need PD” (groups 2 and 4 combined).

The University of Alabama at Birmingham Institutional Review Board (IRB) approval was obtained on May 10, 2019, and a waiver of informed consent was included. The IRB approval number is IRB-160624007 with the study title, *Description of Acute Kidney Injury in Neonates Undergoing Cardiac Surgery with Cardiopulmonary Bypass*. Procedures were followed per the ethical standards of the responsible institutional committee on human experimentation and with the Helsinki Declaration of 1975. The datasets generated during and/or analyzed for this study are available from the corresponding author upon reasonable request.

### Statistical analysis

We identified statistically significant differences in patient characteristics between those who needed PD and those who did not need PD for each of the eras separately (shown in Tables 3 and 6, respectively). Descriptive statistics were performed using percentages for categorical variables and chi-square was used to determine statistically significant differences. Continuous variables that were normally distributed were reported as means with standard deviation and compared using the independent *t*-test, while variables that were not normally distributed were reported as medians with interquartile ranges (IQR) and compared using the Wilcoxon signed-rank test. We used binary logistic regression analysis to determine which variables in Era 1 were associated with the “need for PD.” We used backward selection with step-wise regression to determine the most parsimonious model for predicting the need for PD, incorporating all variables with a *p* < 0.10. All statistical analyses were performed using JMP Pro 16.

**Table 3:** Differences in demographics for those who needed PD vs. did not need PD in Era 1

Era 1				
Variable	Total *N* = 148 (%)	Needed PD *N* = 67 (%)	Did not need PD *N* = 81 (%)	*p*-value (95% CI)
Age at surgery (days)				
Mean ± SD	9.66 ± 6.76	8.44 ± 5.89	10.68 ± 7.29	**0.04**
Median, IQR	7.32 (5.35–11.38)	6.40 (4.49–9.32)	8.31 (5.52–13.96)	(− 0.42 to 0.05)
Sex (male)	92 (62.16)	45 (67.16)	47 (58.02)	0.17
Birthweight (kg)				
Mean ± SD	3.11 ± 0.55	3.02 ± 0.55	3.19 ± 0.54	0.08
Median, IQR	3.09 (2.75–3.46)	3.00 (2.67–3.40)	3.3 (2.88–3.50)	(− 0.35 to 0.02)
Gestational age (weeks)				
Mean ± SD	38.21 ± 1.61	38.04 ± 1.44	38.36 ± 1.76	0.25
Median, IQR	39 (37–39)	39 (37–39)	39 (37.25–39)	(− 0.84 to 0.22)
STAT category				** < 0.01**
1	4 (2.7)	0 (0)	4 (4.94)	
2	7 (4.73)	3 (4.48)	4 (4.94)	
3	30 (20.27)	11 (16.42)	19 (23.46)	
4	74 (50)	29 (43.28)	45 (55.56)	
5	33 (22.3)	24 (35.82)	9 (11.11)	
CPB (min)				
Mean ± SD	114.43 ± 50.65	122.58 ± 49.41	107.79 ± 50.98	0.08
Median, IQR	108.5 (86–133.75)	117 (92–144)	102 (74.5–128)	(− 1.52 to 31.30)
Pre-op SCr (mg/dL)				
Mean ± SD	0.50 ± 0.14	0.53 ± 0.14	0.47 ± 0.14	**0.01**
Median, IQR	0.5 (0.4–0.6)	0.5 (0.4–0.7)	0.5 (0.4–0.6)	(0.01 to 0.11)
Pre-op weight (kg)				
Mean ± SD	3.19 ± 0.55	3.09 ± 0.51	3.28 ± 0.56	**0.04**
Median, IQR	3.20 (2.81–3.50)	3.10 (2.67–3.46)	3.24 (3.00–3.62)	(− 0.36 to − 0.01)
Open chest (yes)	63 (42.57)	42 (62.69)	21 (25.93)	** < 0.01**

All values are *N* (%) except for those with ± which represent mean and standard deviation or median with IQR. *p*-values represent chi-square test for categorical variables and independent *t*-test for continuous variables

Values in bold indicate statistical significance

*PD* Peritoneal dialysis, *STAT* Society of Thoracic Surgeons-European Association for Cardio-Thoracic Surgery, *CPB* Cardiopulmonary bypass, *SCr* Serum creatinine

## Results

### Era 1

In Era 1, 148 neonates who had cardiac surgery requiring CPB met inclusion, and no neonates were excluded from the analysis due to having KST before CPB surgery. Of the 148 neonates in Era 1, 67/148 (45.3%) were classified in the “needed PD” group and 81/148 (54.7%) were classified in the “did not need PD” group (Table 2). Of the 148 neonates, there was only one neonate with > 10% fluid overload. Of the 67 neonates in the “needed PD” group, 9/67 (13.4%) did not have a PD catheter placed in the OR. Of the 81 neonates in the “did not need PD” group, 41/81 (50.6%) had a PD catheter placed in the OR. Demographic data were compared in those who “needed PD” vs. “did not need PD” (Table 3). Bivariate analysis demonstrated that those who needed PD were younger on the day of surgery (median (IQR) = 6.40 (4.49–9.32) days vs. 8.31 (5.52–13.96) days; *p* = 0.04, 95% CI (− 0.42 to − 0.05)) and had a lower pre-operative weight on the day of surgery (mean ± SD = 3.09 ± 0.51 kg vs. 3.28 ± 0.56 kg; *p* = 0.04, 95% CI (− 0.36 to − 0.01)). The lowest median pre-operative SCr was equal for both groups (0.5 mg/dL) but the distribution was a bit different, IQR (IQR = 0.4–0.7 vs. 0.4–0.6; *p* = 0.01, 95% CI (0.01 to 0.11)). The neonates who had an open chest post-operatively were more likely to “need PD” than “did not need PD” (42/63 (66.6%) vs. 21/63 (33.3%); *p* < 0.01). Neonates who “needed PD” had higher STAT scores than those who “did not need PD” (median (IQR) = 4 (4–5) vs. 4 (3–4), *p* < 0.01, 95% CI (0.18 to 0.76)). There were no significant differences in sex, birthweight, gestational age, and CPB time between groups.

Logistic regression analysis showed that pre-operative SCr, pre-operative weight, and open chest post-operatively were independently associated with the need for PD. For every 0.1 mg/dL increase in SCr, the odds of needing PD increased by 36%, controlling for pre-operative weight and open chest post-operatively (AOR 1.36; *p* = 0.02, 95% CI (1.06 to 1.79). Neonates with an open chest post-operatively had 4.73 higher odds of needing PD, controlling for SCr and pre-operative weight (AOR 4.7; *p* < 0.01, 95% CI (2.32 to 9.96)) (Table 4). While pre-operative weight did not reach statistical significance at the level of 0.05 (AOR 0.53; *p* = 0.06, 95% CI (0.26 to 1.04)), we included it as a predictor in our final model for our prospective analysis because it enhanced the ability to predict the need for PD and it met our a priori criteria of *p* < 0.10. Using the prediction equation, we were able to determine the highest AUC for different cutoffs for a neonate with a SCr ≥ 0.8 mg/dL or pre-operative weight ≤ 2.5 kg or having an open chest post-operatively would need prophylactic PD with an AUC of 0.7. These results led us to develop a new PD protocol based on SCr, pre-operative weight, and having an open chest post-operatively (Table 5). Any neonate with a SCr ≥ 0.8 mg/dL and/or pre-operative weight ≤ 2.5 kg and/or have an open chest post-operatively will have a PD catheter placed in the OR during cardiac surgery and dialysis initiated within 4 h of returning to the cardiac ICU.

**Table 4:** Era 1. Backward step-wise logistic regression analysis for “need for PD”

Variable	Crude OR	*p*-value	95% CI	AOR	*p*-value	95% CI
Pre-op SCr (mg/dL)	1.36	**0.01**	1.07 to 1.74	1.36	**0.02**	1.06 to 1.79
Pre-op weight (kg)	0.52	**0.04**	0.27 to 0.96	0.53	0.06	0.26 to 1.04
Open chest (yes)	4.8	** < 0.01**	2.41 to 9.84	4.73	** < 0.01**	2.32 to 9.96

*PD* Peritoneal dialysis, *SCr* Serum creatinine

Values in bold indicate statistical significance

**Table 5:** New PD protocol for PD catheter placement and initiation of PD

Variables
Pre-op SCr ≥ 0.8 mg/dL AND/OR
Pre-op weight ≤ 2.5 kg AND/OR
Open chest

*SCr* Serum creatinine

### Era 2

Era 2 represents our findings after the implementation of our new protocol for PD catheter placement. Demographic data were categorized by the outcome variables “needed PD” and “did not need PD” (Table 6). Of the 97 neonates evaluated, 28/97 (29%) were classified as the “needed PD” group and 69/97 (71%) were classified as the “did not need PD” group. Of the 28 neonates in the “needed PD” group, 28/28 (100%) had the PD catheter placed in the OR. Of the 69 neonates in the “did not need PD” group, only 18/69 (26.1%) had a PD catheter placed (Table 7).

**Table 6:** Differences in demographics for those who needed PD vs. did not need PD in Era 2

Era 2				
Variable	Total *N* = 97 (%)	Needed PD *N* = 28 (%)	Did not need PD *N* = 69 (%)	*p*-value (95% CI)
Age at surgery (days)				
Mean ± SD	7.94 ± 4.67	6.39 ± 2.99	8.57 ± 5.08	**0.05
Median, IQR	7 (5–9.5)	6.5 (5–7.75)	7 (5.5–10.5)	(− 3.83 to − 0.52)
Sex (male)	46 (47.42)	11 (39.29)	35 (50.72)	0.31
Birthweight (kg)				
Mean ± SD	3.09 ± 0.53	3.11 ± 0.44	3.08 ± 056	0.85
Median, IQR	3.04 (2.73–3.45)	3.01(2.82–3.45)	3.05(2.68–3.45)	(− 0.23 to 0.28)
Gestational age (weeks)				
Mean ± SD	38.05 ± 1.33	38.15 ± 1.26	38.01 ± 1.37	**0.63
Median, IQR	39 (37–39)	39 (37–39)	38 (37–39)	(− 0.45 to 0.74)
STAT category				** < 0.01**
1	0 (0)	0 (0)	0 (0)	
2	4 (4.55)	0 (0)	4 (6.45)	
3	14 (15.91)	0 (0)	14 (22.58)	
4	37 (42.05)	8 (30.77)	29 (46.77)	
5	33 (37.5)	18 (69.23)	15 (24.19)	
CPB (min)				
Mean ± SD	121.42 ± 49.25	128.79 ± 35.50	118.43 ± 53.79	**0.08
Median, IQR	118 (89–146)	132 (108.25–156.5)	114 (85–139)	(− 8.22 to 28.93)
Pre-op SCr (mg/dL)				
Mean ± SD	0.53 ± 0.14	0.57 ± 0.14	0.51 ± 0.13	**0.05
Median, IQR	0.51 (0.43–0.6)	0.53 (0.47–0.66)	0.50 (0.41–0.58)	(− 0.01 to 0.12)
Pre-op weight (kg)				
Mean ± SD	3.19 ± 0.53	3.14 ± 0.41	3.22 ± 0.57	**0.41
Median, IQR	3.19 (2.84–3.58)	2.99 (2.89–3.40)	3.27 (2.82–3.61)	(− 0.28 to 0.13)
Open chest (yes)	55 (56.7)	27 (96.43)	28 (40.58)	** < 0.01**

All values are *N* (%) except for those with ± which represent mean and standard deviation or median with IQR. *p*-values represent chi-square test for categorical variables, independent *t*-test for continuous variables, or Wilcoxon signed-rank test indicated by **

Variables in bold indicate statistical significance

*PD* Peritoneal dialysis, *STAT* Society of Thoracic Surgeons-European Association for Cardio-Thoracic Surgery, *CPB* cardiopulmonary bypass, *SCr* Serum creatinine

**Table 7:** 2 × 2 table of Era 2 need for PD by PD catheter placement

	Needed PD	Did not need PD	Total
PD catheter placed (yes)	28 (group 1)	18 (group 2)	46
PD catheter placed (no)	0 (group 3)	51 (group 4)	51
Total	28	69	97

*PD* Peritoneal dialysis

Bivariate analysis demonstrated that neonates with higher STAT scores were associated with the need for PD (median = 5 (4–5) versus 4 (3–4.25), *p* < 0.01, 95% CI (0.52 to 1.09)). The neonates who had an open chest post-operatively were more likely to need PD (*p* < 0.01). There were no significant differences in age at surgery, sex, birthweight, gestational age, CPB time, pre-operative SCr, or pre-operative weight between groups.

In Tables 8 and 9, we show improvement in PD utilization between Era 1 and Era 2. In Era 1, 58/67 (86%) who needed PD had a PD catheter placed in the OR whereas 28/28 (100%) of those who needed PD had a PD catheter placed in the OR in Era 2 (*p* < 0.05) (Table 8). In Era 1, 41/81 (50.1%) who did not need PD had a PD catheter placed in the OR, whereas only 18/69 (26.1%) in Era 2 (*p *< 0.01) (Table 9).

**Table 8:** 2 × 2 table of Era 1 and Era 2 needed PD

	PD catheter placed (yes)	PD catheter placed (no)	Total
Era 1	58	9	67
Era 2	28	0	28
Total	86	9	95

*PD* Peritoneal dialysis

**Table 9:** 2 × 2 table of Era 1 and Era 2 did not need PD

	PD catheter placed (yes)	PD catheter placed (no)	Total
Era 1	41	40	81
Era 2	18	51	69
Total	59	91	150

*PD* Peritoneal dialysis

## Discussion

In this two-part analysis of a retrospective and prospective observational study, we sought to determine which neonates would most benefit from prophylactic PD after CPB. In Era 1, we found three risk factors (SCr ≥ 0.8 mg/dL, pre-operative weight ≤ 2.5 kg, or having an open chest post-operatively) independently associated with the need for PD. Using the characteristics we found in Era 1, we implemented a protocol to determine which neonates would receive prophylactic PD and show improvement in our ability to allocate PD to those who are likely to benefit from PD. Our new PD protocol states that any neonate with a SCr ≥ 0.8 mg/dL and/or pre-operative weight ≤ 2.5 kg and/or have an open chest post-operatively will have a PD catheter placed in the OR during cardiac surgery and dialysis initiated within 4 h of returning to the cardiac ICU. The use of the new protocol decreased the number of neonates who needed PD but did not have a PD catheter placed in the OR from 13.4 to 0% (*p* = 0.05). In addition, we decreased the percentage of neonates who had a PD catheter placed but did not need PD from 50.6 to 26.1% (*p* < 0.01).

The strength of this study is the use of a derivation cohort to determine the characteristics and implementation of these findings into an active clinical protocol. We proactively identify and later validate risk factors associated with the need for PD in neonates who have undergone cardiac surgery requiring CPB. This study has assisted our institution with actively developing a protocol for managing severe AKI post-operatively as the number of neonates who needed PD but did not have a PD catheter placed between Era 1 and Era 2 decreased from 13.4 to 0%, respectively. Proactively identifying these risk factors avoids delays in initiating PD therefore mitigating worsening outcomes including fluid overload, prolonged mechanical ventilation, prolonged ICU length of stay, and mortality.

We believe this protocol will reduce medical expenditures and potential complications associated with PD catheters and PD. We were able to decrease the number of neonates who did not need PD but had a PD catheter placed between Era 1 and Era 2 from 50.6 to 26.1%, respectively. This has important implications in care delivery including resources used and exposing children to therapies that they do not need. While we are pleased to see a significant reduction in the number of PD catheters placed in neonates who did not require PD, we will continue to reevaluate our protocol for continued improvement.

There are some limitations to this study including single-center retrospective analysis and small sample size which is often seen in other pediatric studies. Increasing the sample size can potentially strengthen the study which can then be further validated in larger prospective studies. Another limitation was the screening criterion of > 10% fluid overload used to determine if PD was needed. There was a very limited number of neonates who were > 10% fluid overloaded; therefore, most neonates were categorized as needed PD based on using the PD catheter for > 48 h. Additionally, there is no accepted consensus on when KST should be initiated. Opinions on when to start KST vary between institutions and even among nephrologists at the same institution. Also, there is a lack of consensus for discontinuing KST. This is particularly relevant to our study, as we have utilized the duration of PD as a factor in determining whether PD was necessary. Despite the absence of a widely accepted protocol for discontinuing KST, we had thoughtful discussion and consideration, and our definition of who needs PD was agreed upon by nephrologists, cardiac surgeons, and cardiac intensivists at our institution based on prior experience and previous studies in neonates after CPB.

Acknowledging both the strengths and limitations of our study has allowed us to establish a starting point for standardizing care for these neonates and our data suggest improved resource utilization. Future multi-center prospective studies will further enhance our findings.

## Conclusion

The development and implementation of an evidence-based prophylactic PD protocol improved our ability to determine which neonates are most likely to benefit from prophylactic PD after CPB at our institution. We believe this protocol will improve outcomes and reduce medical expenditures, nursing time, and complications from unnecessary procedures. Assessing these outcomes at our institution will further provide substantial evidence of the benefits of this protocol. Evaluation at other institutions using larger sample sizes may enable universal consensus on which patients can benefit from prophylactic PD in neonates after CPB surgery.

### Supplementary Information

Below is the link to the electronic supplementary material.

Graphical abstract (PPTX 80 KB)
