# Supplementary material for: Deriving and validating a protocol to determine the need for prophylactic peritoneal dialysis in neonates after cardiopulmonary bypass surgery
Source: Pediatr Nephrol. 2024 Mar 4;39(7):2245–51. doi: 10.1007/s00467-024-06327-3 (PMC11147869; doi:10.1007/s00467-024-06327-3)
Supplement: Supplementary file 1 — Graphical abstract (PPTX 80 KB) [file 467_2024_6327_MOESM1_ESM.pptx]

## Slide 1
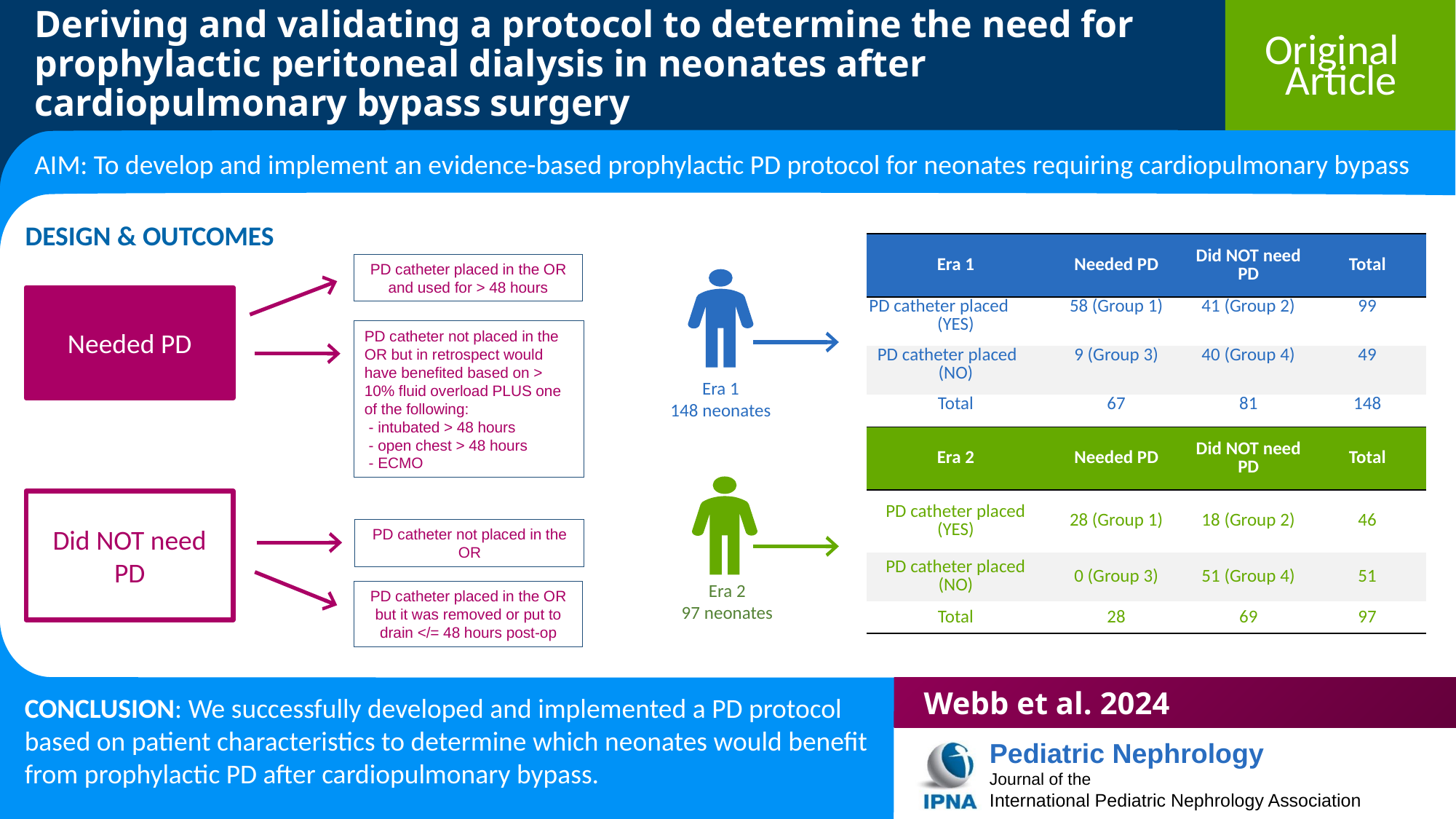

Deriving and validating a protocol to determine the need for prophylactic peritoneal dialysis in neonates after cardiopulmonary bypass surgery
AIM: To develop and implement an evidence-based prophylactic PD protocol for neonates requiring cardiopulmonary bypass
DESIGN & OUTCOMES
| Era 1 | Needed PD | Did NOT need PD | Total |
| --- | --- | --- | --- |
| PD catheter placed (YES) | 58 (Group 1) | 41 (Group 2) | 99 |
| PD catheter placed (NO) | 9 (Group 3) | 40 (Group 4) | 49 |
| Total | 67 | 81 | 148 |
| Era 2 | Needed PD | Did NOT need PD | Total |
| PD catheter placed (YES) | 28 (Group 1) | 18 (Group 2) | 46 |
| PD catheter placed (NO) | 0 (Group 3) | 51 (Group 4) | 51 |
| Total | 28 | 69 | 97 |
PD catheter placed in the OR and used for > 48 hours
Needed PD
PD catheter not placed in the OR but in retrospect would have benefited based on > 10% fluid overload PLUS one of the following:
 - intubated > 48 hours
 - open chest > 48 hours
 - ECMO
Did NOT need PD
PD catheter not placed in the OR
PD catheter placed in the OR but it was removed or put to drain </= 48 hours post-op
Era 1
148 neonates
Era 2
97 neonates
Webb et al. 2024
CONCLUSION: We successfully developed and implemented a PD protocol based on patient characteristics to determine which neonates would benefit from prophylactic PD after cardiopulmonary bypass.
